# Role of *Williamsia* and *Segniliparus* in human infections with the approach taxonomy, cultivation, and identification methods

**DOI:** 10.1186/s12941-021-00416-z

**Published:** 2021-01-23

**Authors:** Mehdi Fatahi-Bafghi

**Affiliations:** Shahid Sadoughi University of Medical Sciences and Health Services, Yazd, Iran

**Keywords:** Actinomycete, Isolation, Taxonomy, *Segniliparus*, *Williamsia*

## Abstract

The genera *Williamsia* and *Segniliparus* are of aerobic actinomycetes and at the time of writing, they have 12 and 2 species, respectively. These genera cause various infections in humans. In this review, we surveyed their taxonomy, isolation, identification, as well as their role to cause human infections.

## Introduction

Aerobic actinomycetes are the group of Gram-positive bacilli belonging to the phylum Actinobacteria. Some species that cause human infections in this group are situated in one of the four suborders, including Corynebacterineae, Micrococcineae, Streptomycineae and Streptosporangineae [1]. Kämpfer et al. and Butler et al. suggested that the genera of *Williamsia* [2] and *Segniliparus* that belong to the actinomycete family, [3] respectively, can cause human infections. They non-spore, non-motile aerobic organisms with short rods without branching that contain mycolic acid components in the cell wall structure [4]. DNA G+C content in the genera *Williamsia* and *Segniliparus* are 64–65% and 68–72% [4] respectively, and they are into the order Corynebacteriales (Tindall [5] proposed the name of *Corynebacteriales* to be replaced by *Mycobacteriales*) and suborder Corynebacterineae. The various genera include *Corynebacterium*, *Dietzia*, *Hoyosella*, *Gordonia*, *Lawsonella*, *Millisia*, *Mycobacterium*, *Mycobacteroides*, *Mycolicibacillus*, *Mycolicibacter*, *Mycolicibacterium*, *Nocardia*, *Rhodococcus*, *Skermania*, *Smaragdicoccus*, *Tomitella* and, *Tsukamurella* are located in this suborder (https://www.ncbi.nlm.nih.gov/Taxonomy/Browser/wwwtax.cgi?mode=Undef&id=85007&lvl=3&lin=f&keep=1&srchmode=1&unlock). To date, various infections cause by actinomycetes are on the rise. The most common genera that cause infections in this suborder include *Corynebacterium* (such as *Corynebacterium accolens* [6], *Corynebacterium afermentans* [6], *Corynebacterium amycolatum* [6], *Corynebacterium appendicis* [7], *Corynebacterium argentoratense* [7], *Corynebacterium aurimucosum* [6], *Corynebacterium coyleae* [7], *Corynebacterium diphtheriae* bv. *mitis* [6], *Corynebacterium durum* [7], *Corynebacterium freneyi* [7], *Corynebacterium glucuronolyticum* [6], *Corynebacterium hansenii* [7], *Corynebacterium imitans* [6], *Corynebacterium jeikeium* [6], *Corynebacterium kroppenstedtii* [8], *Corynebacterium lipophiloflavum* [7], *Corynebacterium macginleyi* [6], *Corynebacterium massiliense* [7], *Corynebacterium minutissimum* [6], *Corynebacterium mucifaciens* [6], *Corynebacterium mycetoides* [7], *Corynebacterium pseudodiphtheriticum* [6], *Corynebacterium pilbarense* [7], *Corynebacterium propinquum* [6], *Corynebacterium pyruviciproducens* [7], *Corynebacterium riegelii* [7], *Corynebacterium resistans* [7], *Corynebacterium simulans* [6], *Corynebacterium singular* [7], *Corynebacterium sputi* [7], *Corynebacterium stationis* [7], *Corynebacterium striatum* [6], *Corynebacterium sundsvallense* [7], *Corynebacterium thomsenii* [7], *Corynebacterium timonense* [7], *Corynebacterium tuberculostearicum* [6], *Corynebacterium tuscaniense* [7], *Corynebacterium ureicelerivorans* [6]), *Gordonia* (such as *Gordonia aichiensis* [9], *Gordonia amicalis* [9], *Gordonia araii* [9], *Gordonia bronchialis* [9], *Gordonia effuse* [9], *Gordonia otitidis* [9], *Gordonia polyisoprenivorans* [9], *Gordonia sputi* [9], *Gordonia terrae* [9], *Gordonia westfalica* [10]), *Mycobacterium* (such as *Mycobacterium abscesus* [11], *Mycobacterium ahvazicum* [12], *Mycobacterium alsense* [13], *Mycobacterium alsiense* [14], *Mycobacterium arupense* [15], *Mycobacterium avium* [11], *Mycobacterium bacteremicum* [14], *Mycobacterium barrassiae* [14], *Mycobacterium bouchedurhonense* [14], *Mycobacterium canettii* [16], *Mycobacterium celeriflavum* [14], *Mycobacterium chelonae* [11], *Mycobacterium chimaera* [17], *Mycobacterium conceptionense* [18], *Mycobacterium engbaekii* [14], *Mycobacterium europaeum *[14], *Mycobacterium flavescens* [19], *Mycobacterium fortuitum* [11], *Mycobacterium fragae* [14], *Mycobacterium franklinii* [14], *Mycobacterium fukienense* [14], *Mycobacterium gadium* [19], *Mycobacterium gordonae* [11], *Mycobacterium heckeshornense* [20], *Mycobacterium heraklionense* [14], *Mycobacterium immunogenum* [21], *Mycobacterium insubricum *[14]*, Mycobacterium intracellulare* [11], *Mycobacterium iranicum* [14], *Mycobacterium malmoense* [22], *Mycobacterium mucogenicum* [11], *Mycobacterium kansasii* [11], *Mycobacterium koreense *[14]*, Mycobacterium kumamotonense* [14], *Mycobacterium kyorinense* [14], *Mycobacterium lentiflavum* [11], *Mycobacterium lepromatosis* [14], *Mycobacterium llatzerense *[14]*, Mycobacterium longobardum* [14], *Mycobacterium mageritense* [23], *Mycobacterium mantenii* [14], *Mycobacterium marinum* [24], *Mycobacterium marseillense* [14], *Mycobacterium monacense* [14], *Mycobacterium novocastrense* [19], *Mycobacterium noviomagense* [14], *Mycobacterium orygis* [14], *Mycobacterium paraffinicum* [14], *Mycobacterium paragordonae* [14], *Mycobacterium parakoreense* [14], *Mycobacterium paraseoulense* [14], *Mycobacterium paraterrae* [14], *Mycobacterium peregrinum* [19], *Mycobacterium porcinum* [19], *Mycobacterium riyadhense* [14], *Mycobacterium scrofulaceum* [11], *Mycobacterium senuense* [14], *Mycobacterium seoulense* [14], *Mycobacterium setense* [14], *Mycobacterium sherrisii* [14], *Mycobacterium shigaense* [14], *Mycobacterium shinjukuense* [14], *Mycobacterium simiae* [25], *Mycobacterium simulans* [14], *Mycobacterium sinense* [14], *Mycobacterium thermoresistibile* [18], *Mycobacterium timonense* [14], *Mycobacterium tuberculosis* [26], *Mycobacterium ulcerans* [27], *Mycobacterium vulneris* [14], *Mycobacterium xenopi* [11], *Mycobacterium yongonense* [14]), *Tsukamurella* (such as *Tsukamurella asaccharolytica* [28], *Tsukamurella conjunctivitidis* [28], *Tsukamurella hongkongensis* [29], *Tsukamurella inchonensis* [29], *Tsukamurella paurometabola* [29], *Tsukamurella pseudospumae* [29], *Tsukamurella pulmonis* [29], *Tsukamurella serpentis* [29], *Tsukamurella sinensis* [29], *Tsukamurella soli* [29], *Tsukamurella spumae* [29], *Tsukamurella sputi* [28], *Tsukamurella strandjordae* [29], *Tsukamurella tyrosinosolvens* [29]), *Nocardia* (such as *Nocardia abscessus* [30], *Nocardia amamiensis* [30], *Nocardia amikacinitolerans* [31], *Nocardia araoensis* [30], *Nocardia arthritidis* [30], *Nocardia asiatica* [30], *Nocardia asteroides* [30], *Nocardia barduliensis* [32], *Nocardia beijingensis* [30], *Nocardia blacklockiae* [33], *Nocardia boironii* [30], *Nocardia brasiliensis* [30], *Nocardia caviae* [30], *Nocardia carnea* [34], *Nocardia cerradoensis* [30], *Nocardia colli* [35], *Nocardia concava* [30], *Nocardia crassostreae* [30], *Nocardia cyriacigeorgica* [30], *Nocardia exalbida* [30], *Nocardia farcinica* [30], *Nocardia gipuzkoensis* [32], *Nocardia harenae* [30], *Nocardia higoensis* [30], *Nocardia ignorata* [36], *Nocardia kruczakiae* [30], *Nocardia mexicana* [30], *Nocardia neocaledoniensis* [30], *Nocardia nova* [30], *Nocardia otitidiscaviarum* [30], *Nocardia paucivorans* [30], *Nocardia pseudobrasiliensis* [30], *Nocardia puris* [30], *Nocardia takedensis* [30], *Nocardia thailandica* [30], *Nocardia transvalensis* [30], *Nocardia veterana* [30], *Nocardia wallacei* [37], *Nocardia yamanashiensis* [30]) and, *Rhodococcus* (such as *Rhodococcus equi* {renamed to *prescottella equi*} [38], *Rhodococcus erythropolis* [38], *Rhodococcus ruber* [38], *Rhodococcus gordoniae* [38], *Rhodococcus facsians* [38]). This study was performed because of the lack of attention and awareness of physicians to infections caused by these bacteria and to inform medical laboratory personnel about the methods of isolation and detection of these bacteria at the genus and species level. Our literature review focused on the human infections caused by *Williamsia* and *Segniliparus* considering taxonomy, cultivation, and identification methods through searching four databases, including Google Scholar, PubMed, Scopus, and Web of Science up to Oct 28, 2020, for all articles in English language, such as case reports, original articles, review article and books were 7, 17, 2, and 3 articles respectively.

## Cell wall structure in *Williamsia*

The genera *Williamsia* and *Segniliparus* has a wall chemotype IV [4, 39]. In the cell wall, *Williamsia* contains meso-2,6-diaminoheptanedioate(C_7_H_14_N_2_O_4_), dihydrogenated menaquinone with nine isoprene units (*Williamsia deligens* has dihydrogenated menaquinone with eight isoprene units [40]), diphosphatidylglycerol, tuberculostearic acids, phosphatidylethanol, phosphatidylglycerol, *N*-glycolyl muramic acid, phosphatidylinositol and, mycolic acids [4]. Muramic acid is glycosylated in the genera of *Tsukamurella*, *Tomitella*, *Smaragdicoccus*, *Skermania*, *Rhodococcus*, *Nocardia*, *Mycobacterium*, *Millisia*, and *Gordonia*, but it is acetylated in the *Dietzia* and* Corynebacterium* [4]. The fatty acids of *Williamsia* are hexadecenoic acid (C16:1-trans) oleic acid (C18:1), palmitic acid (C16:0), and tuberculostearic acid (10-methyl octadecanoate) [4]. In the *Williamsia*, some carbons in chain mycolic acids are C50–C56 [41].

## Cell wall structure in *Segniliparus*

The cell wall of *Segniliparus* contains meso-diaminopimelic acid, mycolic acids and, tuberculostearic acid [3]. The fatty acids of *Segniliparus* are C10:0, C14:0, C16:0, and tuberculostearic acid [3]. In the *Segniliparus*, some carbons in chain mycolic acids are C60–C100 [42], but in the other genera such as *Nocardia*, *Skermania*, *Gordonia*, *Tsukamurella*, *Mycobacterium*, *Millisia*, *Rhodoccocus*, *Dietzia*, *Hoyosella*, and *Corynebacterium* they are C46–C60, C58–C64, C46–C66, C64–C78, C60–C90, C44–C52, C30–C54, C34–C38, C30–C35 and, C22–C36 [41] respectively.

## Isolation methods for *Williamsia* spp.

Collection and transportation of clinical specimens to the medical laboratory are two important principles in the isolation of aerobic actinomycetes from the infections [1]. At the time of writing, the specific media have not been described for the isolation of *Williamsia* from human clinical samples. In literature, various media have been used for *Williamsia* isolation from various sources; however, those associated with good growth or appropriate for morphological examination are columbia agar supplemented with 5% sheep blood agar and brain heart infusion (BHI) agar [40, 43], M3 agar supplemented with cycloheximide and nystatin [44], glucose/yeast extract agar (GYEA) plates [44, 45], raffinose–histidine agar plate supplemented with cycloheximide and nystatin [45], tryptic soy agar (trypticase soy agar/tryptone soy agar) [2, 46–48], starch-casein agar supplemented with cycloheximide [47], nystatin and rifampicin and ISP media 2–7 [47], modified Bennett’s agar [47], glucose-yeast extract malt extract agar [47], nutrient agar [47, 49, 50], Gauze’s medium with cycloheximide, nalidixic acid, novobiocin, and nystatin [51], M125 medium [49], tap water agar and ISP medium 2 [48], Reasoner's 2A agar (R2A) [2], GC agar [52], serum broth [52], and M1 agar plate [53].

## Isolation methods for *Segniliparus* spp.

For the genus *Segniliparus*, the use of Middlebrook 7H10 and 7H11 media [3], Lowenstein–Jensen (LJ) medium [54], LJ with 5% sodium chloride [54] and American Trudeau Society (ATS) media [54] have been suggested for isolation, good growth, and examination of morphological characteristics. Also, *Segniliparus rugosus can* grow on MacConkey agar [3] and it has been reported that *S. rugosus* is resistant to decontamination methods such as NaOH and *N*-acetyl-l-cysteine in clinical specimens [55].

## Phenotypic identification of *Williamsia*

Phenotypic characterizations are the first step for these bacteria identification at the genus and species levels. In the *Williamsia* there is 12 species names validly published includes *Williamsia aurantiacus* [53], *W. deligens* [40], *Williamsia faeni* [51], *Williamsia herbipolensis* [50], *Williamsia limnetica* [47], *Williamsia maris* [44], *Williamsia marianensis* [45], *Williamsia muralis* [2], *Williamsia phyllosphaerae* [49], *Williamsia serinedens* [43], *Williamsia spongiae* [46], and *Williamsia sterculiae* [48]. The species of this genus are distributed in different environments; however, they have also been isolated from clinical specimens [40]. Various phenotypic tests are properties of colonial morphology and pigment production (pigment colors in *Williamsia* spp. are yellow to orange or red [1]), producing aerial hyphae (this phenotypic characterization is seen in *Williamsia*, *Skermania*, *Nocardia* and, *Millisia* [4]), Gram stain (the genus *Williamsia* is Gram-positive), acid-fast stain (the genus *Williamsia* is not acid-fast [56]), hydrolysis of amino acids, acid production of carbohydrates, high-performance liquid chromatography **(**HPLC), gas–liquid chromatography (GLC), thin-layer chromatography (TLC) procedures, and enzymes production [3, 4, 39, 56]. Some of the phenotypic characterization of the *Williamsia* spp. are shown in Table 1. Conventional phenotypic methods are unreliable and insufficient for differentiation of *Williamsia* and *Segniliparus* of related aerobic actinomycetes; therefore, molecular techniques have been used for accurate identification at the genus and species level. The temperature range for the growth for *Williamsia* spp. including *W. limnetica*, *W. sterculiae*, *W. maris*, *W. muralis* is 10 to 37 °C [2, 44, 47, 48], for *W. aurantiacus* and *W. spongiae* is 10 to 45 °C [46, 53], for *W. phyllosphaerae* and *W. herbipolensis* is 25 to 30 °C [49, 50], for *W. deligens* is 37 °C [40], for *W. serinedens* is 22 to 30 °C [43], for *W. marianensis* is 4 to 30 °C [45], and for *W. faeni* is 10 to 30 °C [51]. Also, the pH range for the growth is between 4.0 and 10.0 for *W. aurantiacus* and *W. spongiae* [46, 53] and 5.0–8.0 for *W. sterculiae* [48].

**Table 1:** Some of the phenotypic characterization of the *Williamsia* and *Segniliparus* spp.

Name of bacteria	Source isolation/year	Utilization of													
		Adonitol	d-Mannitol	d-Mannose	meso-Inositol	d-Trehalose	d-Xylose	l-Fructose	d-Glucose	d-Sorbitol	d-Sucrose	l-Arabinose	d-Cellobiose	d-Maltose	d-Melibiose
*W. aurantiacus*	Marine sponge/2019	−	−	+w	−		−	+		−		−	−	−	
*W. deligens*	Human blood/2006	−			−							−		+	
*W. faeni*	Hay meadow/2010	+	+		+	+	+	+	+	+	+	+	+	+	+
*W. herbipolensis*	Phyllosphere of *Arabidopsis thaliana*/2016	−	+	+		w+	w+	+	+	+	+	−	−	−	−
*W. limnetica*	Sediment/2012	−	+	+	−		+	+	+	−	+	−	+	+	+
*W. marianensis*	Sediment/2008	−	+	+	−	+	+			+	+	+		−	
*W. maris*	Sediment/2004	−	+	−	+	+	+	+	+	+	+	−	−	−	−
*W. muralis*	Indoor building materials/1999	+	+	+				+	+	+	+				
*W. phyllosphaerae*	The leaf surface of *Trifolium repens*/2011	−	+	−		−	−	+	+	+	+	−	−	−	−
*W. serinedens*	Oil-contaminated soil/2007	+			−	+	+			+	+	+		+	
*W. spongiae*	Marine sponge/2017	−	−	−	−			+		−		−	−		
*W. sterculiae*	Stems of medicinal plants/2013				+		+		+	+	+				
*S. rugosus*	Sputum/2005		+/−					−		+/−	−				
*S. rotundus*	Sputum/2005		−					+		−	+/−				

Name of bacteria	Source isolation/year	Utilization of							Production of urease	Hydrolysis of gelatin	Hydrolysis of casein	Hydrolysis of hypoxanthine	Hydrolysis of tyrosine	References
		l-Rhamnose	l-Sorbose	l-Arabitol	d-Galactose	d-Raffinose	d-Ribose	d-Salicin						
*W. aurantiacus*	Marine sponge/2019	−			−	−	−				+	+		[53]
*W. deligens*	Human blood/2006	−			−				+	−	−	−	−	[40]
*W. faeni*	Hay meadow/2010	+		+	+	+	+	−	+	+				[51]
*W. herbipolensis*	Phyllosphere of Arabidopsis thaliana/2016	−			−		−	−						[50]
*W. limnetica*	Sediment/2012	+	−		+	−				−	−	−		[47]
*W. marianensis*	Sediment/2008	+			−	−		−		−	+	+	−	[45]
*W. maris*	Sediment/2004	+	+	−	−	−	−	−						[44]
*W. muralis*	Indoor building materials/1999	+												[2]
*W. phyllosphaerae*	The leaf surface of *Trifolium repens*/2011	−			−		−	−						[49]
*W. serinedens*	Oil-contaminated soil/2007	−			+				+	+	−	−	−	[43]
*W. spongiae*	Marine sponge/2017	−			−	−	+			−	+	+		[46]
*W. sterculiae*	Stems of medicinal plants/2013	+		+					+	+			−	[48]
*S. rugosus*	Sputum/2005													[3]
*S. rotundus*	Sputum/2005													[3]

## Phenotypic identification of *Segniliparus*

Two species of *Segniliparus rotundus* and *S. rugosus* belong to the *Segniliparus* genus [3]*.* Its species are distributed in different environments; however, they have also been isolated from clinical specimens [57]. Pigment colors in *Segniliparus* spp. is white to beige [3]. An aerial hyphae are not seen in the *Segniliparus* [4]; and the genus is acid-fast [56]. Some of the phenotypic characterization of the *Segniliparus* spp. are shown in Table 1. The temperature range for the growth in *Segniliparus* spp. are as follows: *S. rotundus*: 28 to 37 °C [3] and *S. rugosus*: 22 to 42 °C [3]. Researchers, medical laboratory personnel, and clinicians should note that in pulmonary specimens, especially in cystic fibrosis patients, the genus *Segniliparus* is similar to the genus *Mycobacterium* in acid‐fast staining [54].

## Molecular identification of the *Williamsia*

**Fig. 1:**
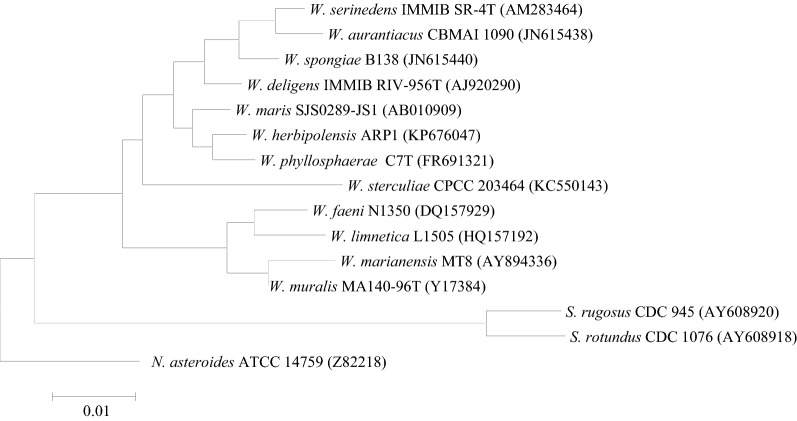
The 16S rRNA gene-based phylogenetic tree of standard *Williamsia* and *Segniliparus* spp. with using the molecular evolutionary genetics analysis (MEGA) 5.0 software [66] which computed by the neighbor joining (NJ) analyses and kimura 2-parameter (K2P) model. The sequences were downloaded from NCBI. W: *Williamsia*, N: *Nocardia*, S: *Segniliparus*

The most common molecular method for *Williamsia* accurate identification and assessment of taxonomic characteristics is sequence-based identification. 16S rRNA gene sequencing is an effective standard method for accurate identification of the novel bacteria and emerging pathogens at the genus and species levels [58]. Primers to amplify 16S rRNA gene for *Williamsia* identification include 27F (5′-AGAGTTTGATCCTGGCTCAG-3′)/1492R (5′-GGTTACCTTGTTACGACTT-3′) and 27f (5′-GAGTM′GATCCTGGCTCAG-3′)/1525r (5′-AGAAAGGAGGTGATCCAGCC-3′) [40, 44]. Montoya-Porras et al. [59] identified the genus *Williamsia* with 454 pyrosequencing for the variable region of the 16S rRNA gene. The phylogenetic tree of the 16S rRNA gene for *Williamsia* standard species is shown in Fig. 1. The gold standard method to discern bacterial species is DNA–DNA hybridization (DDH) [30]; however, this method is not used in clinical laboratories for bacterial identification. Another molecular method is the whole-genome sequencing (WGS), which has been deposited for five *Williamsia* species in the National Center for Biotechnology Information (NCBI). Data are provided in Additional file 1.

## Molecular identification of the *Segniliparus*

16S rRNA gene primers, such as 5′-GAGAGTTTGATCCTGGCTCAG-3′/5′-AAGGAGGTGATCCAGCCGCA-3′ [3]; 8FPL (5′-AGTTTGATCCTGGCTCAG-3′)/806R (5′-GGACTACCAGGGTATCTAAT-3′), and 515FPL (5′-TGCCAGCAGCCGCGGTAA-3′)/13B (5′-AGGCCCGGGAACGTATTCAC-3′) have been used for *Segniliparus* identification [60]. The phylogenetic tree of the 16S rRNA gene for *Segniliparus* standard species is shown in Fig. 1. Butler et al. [3] reported that three of the four isolates of *Segniliparus* were not amplified for the 65 kDa heat-shock protein (*hsp65* gene) with TB11 (5′-ACCAACGATGGTGTGTCCAT-3′) and TB12 (5′-CTTGTCGAACCGCATACCCT-3′) primers. Also, the cholesterol oxidase gene (*choE* gene) (this gene is a virulence factor gene in *Rhodococcus equi*) was not amplified for *Segniliparus* [3]. Koh et al. [55] used PCR-restriction fragment length polymorphism analysis (PRA) of the *hsp65* (527-bp) [F: 5′-GAGGGCGTCATCACCGTCGAGG-3′/R: 5′-CGGCGATGGCGTCGGAGTCACC-3′] and *rpoB* (360-bp) [F: 5′-TCAAGGAGAAGCGCTACG A-3′/R: 5′-GGATGTTGATCAGGGTCTGC-3′] genes for *Segniliparus* spp. identification. WGS of two *Segniliparus* isolates has been deposited in the National Center for Biotechnology Information (NCBI). Data are provided in Additional file 1.

## Pathogenesis in *Williamsia* and *Segniliparus*

Our knowledge about pathogenesis and virulence factors in two genera is limited. Cell wall components, such as mycolic acid, phagolysosome inhibition, immune response promotes, and the production of enzymes, such as catalase, may play a role in their pathogenesis.

## Clinical disease, antibiogram and treatment associated with *Williamsia*

Physicians need to pay attention to these symptoms such as bilateral alveolar infiltrates [61], fever [62], having an underlying disease such as diabetes mellitus for detection of this rare infection [52]. Infections in humans caused by *Williamsia* have been reported. Infection occurs as a result of exposure to the environment; however, there is no evidence of an environmental source for *Williamsia* and *Segniliparus* infections. For antimicrobial susceptibility testing (AST), breakpoints have not been established for these genera, and researchers use recommended AST (the gold standard for antibiogram is micro broth dilution) for *Nocardia* and related aerobic actinomycetes by the Clinical and Laboratory Standards Institute (CLSI) [63]. Tomas et al. [61] first reported *W. muralis* as the cause of lung infection in an old woman. In their study, this bacterium was isolated from a brush sample and results of AST showed that this bacterium was susceptible to amoxicillin-clavulanate, cephalosporin (cefotaxime), carbapenem (imipenem), Quinolone (ciprofloxacin), aminoglycoside (tobramycin, gentamicin), sulfonamide (cotrimoxazole) and resistant to beta-lactam (ampicillin) and macrolide (erythromycin) family. In another study by Yassin et al. [40] reported *W. deligens* of human blood in 2006. Also, *W. serinedens* has been isolated of perinatal sepsis from a pregnant woman in 2010 and this bacterium was susceptible to amikacin, ampicillin, doxycycline, imipenem, linezolid, meropenem, penicillin G, tobramycin, vancomycin and was resistant to oxacillin and trimethoprim-sulfamethoxazole with E-test method [62]. The case reports published regarding *Williamsia* spp. are provided in Table 2.

**Table 2:** Case reports published of *Williamsia* spp. in literature

Age/sex/country	Underlying disease	Type of infection	Isolated from	Name of organism	Outcome	References
66/M/Australia	Diabetic	Endophthalmitis	Vitreous fluid	*W. muralis*	Cure	[52]
80/F/Spain	Allergy to penicillin and high blood pressure	Lung infection	Brush	*W. muralis*	Died	[61]
31/F/Germany	Pregnant	Perinatal sepsis	Blood	*W. serinedens*	Cure	[62]

*W. deligens* isolated from blood [40] but case history is not available

## Clinical disease, antibiogram and treatment associated with *Segniliparus*

Physicians should pay more attention to symptoms such as chronic cough and sputum more than 3 months, fever, multiple small nodules in lung [55, 60] and radiologic finding similar to other genera in actinomycete family such as *M. tuberculosis*, non-tuberculous mycobacteria (NTM), and *Nocardia* for detection of this rare infection. Several studies have reported infections in humans caused by *Segniliparus*. The first report of the *Segniliparus* isolation from the clinical sample was published in 2005 by Butler et al. They isolated *S. rugosus* and *S. rotundus* from sputum and was AST performed using micro broth dilution. The results of AST showed that *S. rotundus* was susceptible to amikacin, cefoxitin, clarithromycin, ciprofloxacin, doxycycline, imipenem, sulfamethoxazole, and *S. rugosus* was susceptible to amikacin and sulfamethoxazole and resistant to clarithromycin, doxycycline, and tobramycin [3]. Butler et al. [64] isolated *S. rugosus* from 3 cystic fibrosis patients in 2007. Moreover, a study by Hansen et al. [54] isolated *S. rugosus* from sputum in a female with cystic fibrosis, and AST using micro broth dilution showed that this isolate was susceptible to ciprofloxacin, gatifloxacin, imipenem and resistant to amikacin, cefoxitin, ceftriaxone, tobramycin. Koh et al. [55] isolated *S. rotundus* from sputum in 2011 in a patient treated with clarithromycin and ciprofloxacin. In another study, *S. rugosus* was isolated from sputum in 2014 [60]. *S. rugosus* possibly is an emerging pathogen in cystic fibrosis patients. Antibiotic resistance genes have not been reported in the genera *Williamsia* and *Segniliparus* [65]. The case reports published on *Segniliparus* spp. are provided in Table 3. On the basis of the clinical reports, the pulmonary infection of *Segniliparus* spp. is associated with chronic cough, fever and hypoventilation, as well as the presence of multiple small nodules, with symptoms of acid-fast bacilli in sputum and radiologic results similar to *M. tuberculosis,* NTM, *Nocardia* and so on. Therefore, pulmonary infection should be identified in microbiology laboratories.

**Table 3:** Case reports published of *Segniliparus* spp. in literature

Age/sex/country	Underlying disease	Type of infection	Isolated from	Name of organism	Outcome	References
/M/USA	Cystic fibrosis	Lung infection	Sputum	*S. rugosus*	Cure	[64]
/M/USA	Cystic fibrosis	Lung infection	Sputum	*S. rugosus*	Cure	[64]
28/M/USA	Cystic fibrosis	Lung infection	BAL	*S. rugosus*	Cure	[64]
Teenager/F/Australia	Cystic fibrosis	Lung infection	Sputum	*S. rugosus*		[54]
43/F/South Korea	Immunocompetent	Lung infection	Sputum	*S. rugosus*	Cure	[55]
47/F/Korea	Immunocompetent	Lung infection	Sputum	*S. rugosus*	Cure	[60]

*Segniliparus rotundus* and *Segniliparus rugosus* isolated from sputum [3] but case history is not available

## Conclusion

In this review, we surveyed taxonomy and the role of the genera *Williamsia* and *Segniliparus* in human infections. The identification of pathogenic factors in these bacteria requires more investigations. A few studies have been conducted on *Williams* and *Segniliparus* infections because of the lack of attention and insufficient experience in medical laboratory personnel as well as the lack of optimization of the phenotypic and molecular methods to identify these bacteria in hospitals. The use of novel molecular methods is necessary for accurate identification of *Williamsia* and *Segniliparus* species.

## Supplementary Information

**Additional file 1. **Whole genome sequence data of *Williamsia* and *Segniliparus* spp.
