# Supplementary material for: Role of Williamsia and Segniliparus in human infections with the approach taxonomy, cultivation, and identification methods
Source: Ann Clin Microbiol Antimicrob. 2021 Jan 23;20:10. doi: 10.1186/s12941-021-00416-z (PMC7825236; doi:10.1186/s12941-021-00416-z)
Supplement: Supplementary file 1 — Additional file 1. Whole genome sequence data of Williamsia and Segniliparus spp. [file 12941_2021_416_MOESM1_ESM.docx]

**Whole genome sequence data of *Williamsia* and *Segniliparus* spp.**

| **Name species/strain** | **Accession number** | **Size(bp)** | **CDSs(total)** | **Genes(coding)** |
| --- | --- | --- | --- | --- |
| *W. muralis* NBRC 105860 | NZ_BDAP00000000 | 5538168 | 5,084 | 4,995 |
| *W. sterculiae* CPCC 203464 | NZ_FTNT00000000 | 4440998 | 4,095 | 4,007 |
| *W. herbipolensis* ARP1 | NZ_JXYP00000000 | 4744957 | 4,425 | 4,326 |
| *W. muralis* DSM 44343 | NZ_RBKV00000000 | 5401534 | 4,943 | 4,867 |
| *W. marianensis* DSM 44944 | NZ_QEOM00000000 | 5568863 | 5,161 | 5,038 |
| *W. limnetica* DSM 45521 | NZ_QJSP00000000 | 6353307 | 5,942 | 5,830 |
| *W. marianensis* BULT 1.1 | NZ_PEBD00000000 | 5233905 | 4,769 | 4,683 |
| *Williamsia* sp. Leaf354 | NZ_LMPL00000000 | 4566644 | 4,180 | 4,133 |
| *Williamsia* sp. D3 | NZ_AYTE00000000 | 5623123 | 5,272 | 4,854 |
| *Williamsia* sp. 1138 | NZ_MJEJ00000000 | 6080896 | 5,592 | 5,494 |
| *Williamsia* sp. 1135 | NZ_MJEI00000000 | 5981226 | 5,581 | 5,350 |
| *S. rotundus* DSM 44985 | NC_014168 | 3157527 | 3,032 | 2,942 |
| *S. rugosus* ATCC BAA-974 | NZ_ACZI00000000 | 3587529 | 3,461 | 3,337 |
